# Neurophysiological correlates of perception–action binding in the somatosensory system

**DOI:** 10.1038/s41598-020-71779-0

**Published:** 2020-09-09

**Authors:** Julia Friedrich, Julius Verrel, Maximilian Kleimaker, Alexander Münchau, Christian Beste, Tobias Bäumer

**Affiliations:** 1 Cognitive Neurophysiology, Department of Child and Adolescent Psychiatry, Faculty of Medicine of the TU Dresden, Schubertstrasse 42, 01309 Dresden, Germany; 2 Institute of Systems Motor Science, University of Lübeck, Lübeck, Germany; 3 Department of Neurology, University Hospital Schleswig-Holstein, Campus Lübeck, Lübeck, Germany

**Keywords:** Neuroscience, Psychology

## Abstract

Action control requires precisely and flexibly linking sensory input and motor output. This is true for both, visuo-motor and somatosensory-motor integration. However, while perception–action integration has been extensively investigated for the visual modality, data on how somatosensory and action-related information is associated are scarce. We use the Theory of Event Coding (TEC) as a framework to investigate perception–action integration in the somatosensory-motor domain. Based on studies examining the neural mechanisms underlying stimulus–response binding in the visuo-motor domain, the current study investigates binding mechanisms in the somatosensory-motor domain using EEG signal decomposition and source localization analyses. The present study clearly demonstrates binding between somatosensory stimulus and response features. Importantly, repetition benefits but no repetition costs are evident in the somatosensory modality, which differs from findings in the visual domain. EEG signal decomposition indicates that response selection mechanisms, rather than stimulus-related processes, account for the behavioral binding effects. This modulation is associated with activation differences in the left superior parietal cortex (BA 7), an important relay of sensorimotor integration.

## Introduction

In everyday life, performing actions is based on relating sensory information to motor output. Whether you grab a cup or open a car door, the proper association/binding of stimuli and actions is an essential prerequisite to control actions. This encompasses and requires the integration of both visuo-motor and somatosensory-motor information.

The Theory of Event Coding (TEC) provides a comprehensive cognitive framework for perception–action integration. According to TEC, features defining a stimulus are combined in ’object files’, whereas features related to an action are stored in ’action files’^1^. Combining object and action files, ’event files’ comprise all stimulus- and response-related features as well as the links/associations that are formed between stimulus and response features^2,3^. An event file can therefore be regarded as a network that stores binding information between the stimulus- and response-related features. Its activation follows a pattern-completion logic: once an element (i.e., a single stimulus or response feature) of the event file is re-encountered, activation automatically spreads to other elements reactivating the whole event file^3–5^. Consequently, responding to a given sensory information depends on previously established sensory-motor associations. For instance, if the same (or similar) sensory information requires different motor responses in consecutive actions, the stimulus–response binding (event file) has to be adjusted, a process that usually leads to higher error rates and longer response times^6,7^. On the other hand, no adjustment is needed when the same stimulus features require the same response, resulting in a performance benefit^8^.

Stimulus–response binding occurs automatically whenever stimuli and responses appear in close temporal proximity^9^. It is easier to form a new event file in case all or no features of the previously stored event file are repeated. The formation of a new event file is hampered when only some of the features overlap as compared to all or none indicating the necessity for reconfiguration of the already stored information^1^. This is also referred to as ’partial-repetition cost’ and indicates temporal binding of stimulus and response features^3^. Thus, stimulus feature repetition improves task performance when the response is repeated (repetition benefits)^8,10^. Correspondingly, feature repetition should deteriorate performance in case the response is alternated (repetition costs)^11,12^. Often, a stimulus–response task (S-R task) is used to investigate stimulus–response binding^8^. In its original form, two visual stimuli are presented successively which are defined by the same (three) stimulus dimensions (e.g., varying in orientation, position, and color). Prior to the presentation of the stimuli, a cue is given requiring a binary choice as soon as the first stimulus (S1) appears. This response, which is independent of the features of S1, serves to establish an association between stimulus and response features in an experimentally controlled way on a trial-by-trial basis. The second stimulus (S2) also requires a binary choice, depending on the features of S2, so that the interference between repeating or alternating stimulus and response features can be measured.

Until now, the neural mechanisms underlying event file binding effects have extensively been investigated in visuo-motor paradigms using EEG and fMRI methods^13–18^. These studies support central aspects of TEC^11,12^ using different types of stimuli like pictures of objects and faces requiring different actions (e.g., finger or facial responses). A study considering the TEC investigated the neurophysiological basis of distractor-response binding mechanisms in the visual domain using letter stimuli and found that event files but not object or action files are modulated by stimulus–response interactions^11^. This was corroborated in a recent study examining neurophysiological mechanisms of event file binding in the visual domain using the S-R task comprising vertical and horizontal bars of different colors^8^. The authors applied different neurophysiological methods, including event-related potential (ERP) analysis and EEG signal decomposition^12^.

It is an open question, though, whether the neural mechanisms underlying visuo-motor event file coding differ from those underlying the somatosensory-motor processing. This is a non-trivial question because the somatosensory (triggered by air puff stimuli) and visual modalities differ in their potential to trigger response inhibition^19^, which is also considered an action that can be bound to a specific stimulus^1,17^. Moreover, the visual and somatosensory domains seem to differ regarding their susceptibility to interference during response inhibition^20^. Sensory lateral inhibition processes, as well as differences in area-specific processing and developmental factors have been found to modify executive functioning based on somatosensory input^21–24^. Furthermore, different stimulus magnitudes have been demonstrated to impact action control presumably due to modulations in the strength of event file binding^25^. Aforementioned studies used vibro-tactile stimuli to trigger response inhibition in a Go/Nogo paradigm.

In the present study, a somatosensory-motor S-R task was conducted using electro-tactile stimuli. The detailed task description can be found in the “Materials and methods” section. According to the visual task explained above, a single electrical pulse was used as a cue, whereas stimuli one (S1) and two (S2) differed with regard to two dimensions: stimulation site (i.e., thumb or little finger) and applied pulse number (double or four pulses). Accordingly, finger compatibility means that electrical stimulation of S1 and S2 was applied to the same finger. Pulse compatibility in turn refers to the application of the same pulse number. Using this task design, the effects of repeating all, one or no stimulus features as well as response repetition or alternation can be investigated (i.e., repetition benefits and costs). Repetition benefits/costs are indicative of a temporal binding of stimulus- and response-related features^2,3,5^. Due to the above-mentioned differences between the modalities with respect to response inhibition, it can be assumed that binding processes also differ with regard to other types of actions. However, based on the current state of knowledge, no specific hypotheses can be put forward regarding the extent to which visual and somatosensory modalities differ in terms of repetition benefits or costs. The reason is that both somatosensory-motor and visuo-motor binding processes play a role in everyday life to coordinate actions properly. Based on this point, binding processes in both modalities have the potential to interfere with or contribute to the selection of appropriate actions.

To improve understanding of potential differences between somatosensory-motor and visuo-motor binding underlying neurophysiological processes were investigated using EEG. It provides the optimal tool since the EEG signal allows the extraction of neurophysiological correlates underlying observed behavioral effects. However, it has to be taken into account that standard ERP components like the N2 or P3 are a composite of various signals originating from different neural sources. The P3 was found to reflect stimulus- and response-related processes^26^ and the N2 is assumed to comprise information related to perception of a stimulus as well as response selection^27,28^. To disentangle neurophysiological processes, the EEG signal has to be decomposed^29^. This can be achieved by the RIDE algorithm that decomposes components into clusters, taking into account inter- and intra-individual variability in processing latency^29,30^. The result is a stimulus- and a response-locked cluster (S- and R-cluster, respectively) as well as an intermediate C-cluster with variable latency occurring between stimulus and response^31^. Processes linked to stimulus perception and encoding are assumed to be reflected by the S-cluster whereas the R-cluster is associated with preparing and executing a response. Processes occurring in between including evaluating stimulus input or translating the stimulus into a specific response are assumed to be covered by the C-cluster^26,29,32,33^.

It is noteworthy that there is an overlap between the RIDE clusters and above-described TEC concepts^11,12^. While the stimulus-related object file might be reflected by the S-cluster, action-related features are more likely to be displayed in the R-cluster. Processes reflecting stimulus–response transition encoded in event files are likely allocated to the C-cluster assumed to reflect intermediate processes occurring between stimulus and action. Modulations in the C-cluster have indeed been found to underlie stimulus–response binding^11,12^. Based on the assumption that event files represent the connection between stimulus and response features and the observation that this binding is reflected by central stimulus–response transition processes, it is hypothesized that the C-cluster is modulated by stimulus–response binding during somatosensory-motor processing akin to previous studies on action control using somatosensory stimuli^20,22,23,25^.

However, stimulus-related processes as reflected by the S-cluster have also been found to be modulated in this context^34^. For example, the P2 component has already been found to be evident in the S-cluster time window^34^ and has repeatedly been linked to the allocation of processing resources^35,36^. Based on these findings, it seems reasonable to investigate whether effects are modulated by differences in attentional processing of different compatibility conditions (i.e., repetition or alternation of stimulus features as well as the response). The analysis of P1 and N1 components was not conducted since it has repeatedly been demonstrated that, although experimental manipulations were performed on a sensory level (i.e., by modulating processing at the somatosensory level or directly varying stimulus input), effects manifested beyond pure perceptual processing and were evident on the response selection level^20,22,23,25^. Since P1 and N1 components constitute early electrophysiological responses to sensory stimulus modulations, stimulus–response association processes were not expected to be reflected by these components. Also, previous findings on event file binding processes did not show modulations in these ERP-components^12^. This is in line with theoretical conceptions of TEC stating that perceptual properties of stimuli are coded in object files and not in event files^3^ which are focus of the current study.

Taken together, event file binding mechanisms are assumed to predominantly modulate processes in the P3 time window associated with stimulus–response transition. Yet, especially in the P3 time window, components with variable latency are prominent^29–31^. Therefore, results paralleling behavioral findings are only expected based on RIDE decomposed data. This notwithstanding, analysis of standard ERPs like P2 and P3 is still conducted since the amount of distortion created by averaging ERPs varies with the degree of latency variability of the modulated component^29,30^. Furthermore, it is possible to evaluate the benefit of RIDE application by comparing it to the results for standard ERPs.

## Results

### Behavioral data

**Figure 1:**
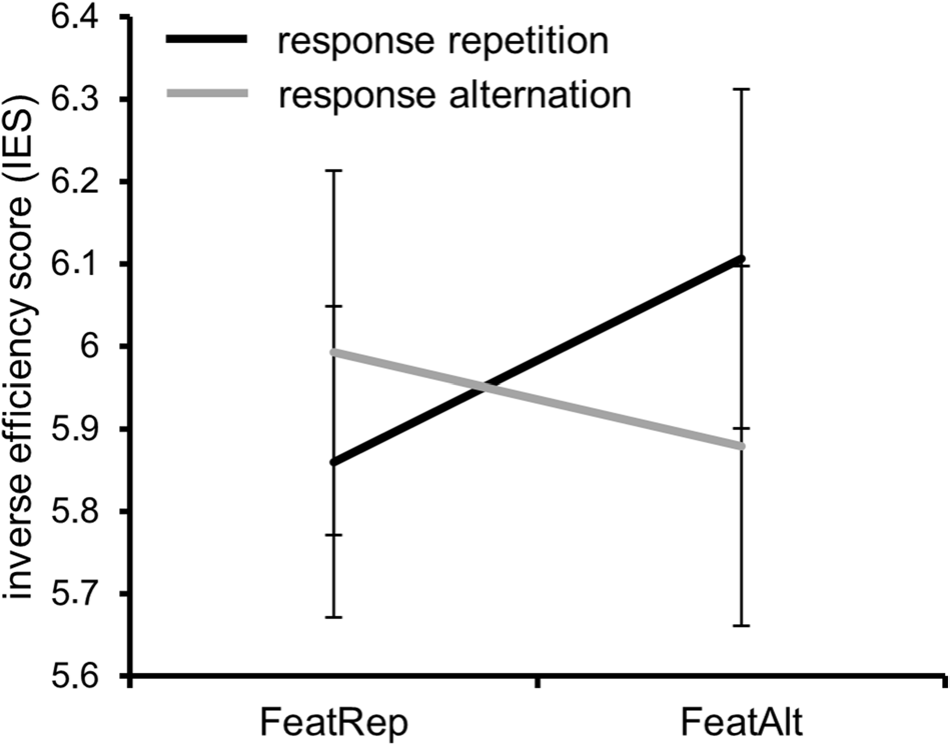
Behavioral data (inverse efficiency index) showing the interaction of “response x finger compatibility”. The IES is illustrated in the feature (finger) repetition (FeatRep) condition and the feature (finger) alternation (FeatAlt) condition for response repetition and response alternation. Error bars indicate standard error of the mean.

Three within-subject factors were defined for behavioral analyses. One factor constitutes “feature (finger) compatibility” describing whether stimulation was delivered to the same finger twice (i.e., feature repetition) or to alternating fingers (i.e., feature alternation). “Pulse compatibility” as another factor defines whether the stimulation encompassed the same or alternating pulse sequence. The factor “response” describes whether the same (response repetition) or different responses (alternation) were required. The repeated measures ANOVA conducted for accuracy rates revealed no significant main or interaction effects (all F ≤ 3.56; p ≥ 0.07). Analysis of reaction times showed a significant main effect of “pulse compatibility” [F(1,27) = 4.34; p = 0.047; *η*_*p*_^2^ = 0.138] and an interaction of “response x finger compatibility” [F(1,27) = 9.65; p = 0.004; *η*_*p*_^2^ = .263]. The interaction of “response x finger compatibility” is of central importance, since it demonstrates the occurrence of stimulus–response binding^12^. Since stimulus–response binding is investigated in the present study, this interaction should be focused on with regard to behavioral and neurophysiological data. Post-hoc paired t-tests revealed that stimulating the same finger repeatedly versus alternating the stimulation site resulted in significantly different reaction times when the response was repeated [t(27) = -3.58; p = 0.001] but not when the response was alternated [t(27) = 0.90; p = 0.374]. In case of response repetition, responses were faster when the same finger was repeatedly stimulated (555 ms ± 18) than when it was alternated (573 ms ± 19). Furthermore, post-hoc paired t-tests showed that response repetition and alternation did not differ when stimulation was repeated at the same finger [t(27) = − 1.42; p = 0.168] but when the finger was alternated [t(27) = 2.12; p = 0.043]. Responses were faster when the response was alternated (558 ms ± 17) than when it was repeated (573 ms ± 19). Since results regarding the interaction effect of a repetition or alternation of response and finger stimulation differed between accuracy rates and reaction times, an “inverse efficiency score” (IES) was computed. It allows to calculate a single performance measure combining reaction time and accuracy rates by forming the ratio of these two measures^37^. It is therefore useful to account for a speed-accuracy trade-off^38^. Smaller IES values indicate superior performance. The repeated measures ANOVA revealed a main effect of “pulse compatibility” [F(1,27) = 4.52; p = 0.043; *η*_*p*_^2^ = 0.143] and an interaction of “response x finger compatibility” [F(1,27) = 9.56; p = 0.005; *η*_*p*_^2^ = 0.261]. Post-hoc paired t-tests again revealed a significant difference between repetition and alternation of finger stimulation when the response was the same [t(27) = − 3.06; p = 0.005], but not when the response was alternated [t(27) = 1.18; p = 0.248]. For repeated responses, performance was superior when the finger was repeatedly stimulated compared to the condition when finger stimulation was alternated. This indicates that participants showed repetition benefits but no repetition costs in the tactile event file paradigm. The interaction is illustrated in Fig. 1. Post-hoc paired t-tests also revealed that response repetition and alternation conditions neither differed when the same finger was repeatedly stimulated [t(27) = − 1.27; p = 0.216], nor when the stimulated finger alternated [t(27) = 1.47; p = 0.152].

### Standard event-related potentials (ERP components)

**Figure 2:**
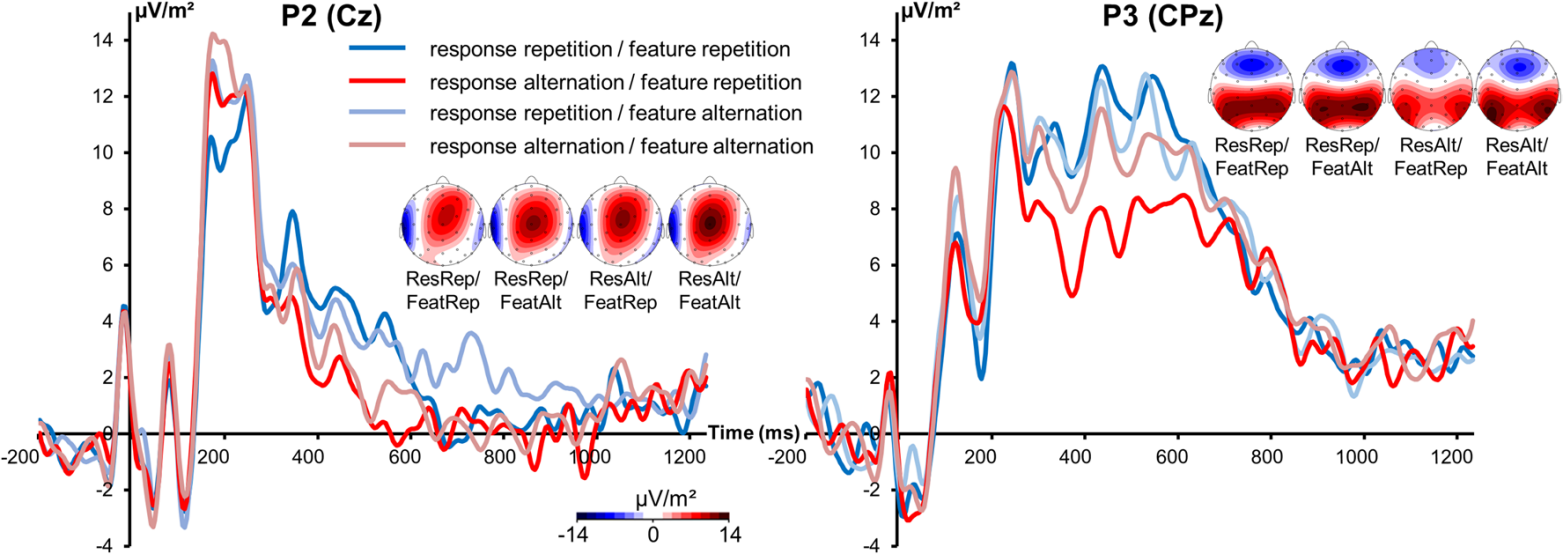
The P2 component at electrode Cz (left) and the P3 component at electrode CPz (right). Time point 0 marks the onset of S2 stimulus presentation. Different colors of the components represent different conditions (ResRep/FeatRep = response repetition/feature (finger) repetition; ResRep/FeatAlt = response repetition/feature (finger) alternation; ResAlt/FeatRep = response alternation/feature (finger) repetition; ResAlt/FeatAlt = response alternation/feature (finger) alternation) according to the legend. Scalp topography plots show the P2 and P3 component in the different conditions with red and blue indicating positive and negative values, respectively.

The P2 and P3 ERP components are illustrated in Fig. 2.

The repeated measures ANOVA of the P2 component at electrode Cz revealed a main effect of “response” [F(1,27) = 8.36; p = 0.007; *η*_*p*_^2^ = 0.236]. Larger amplitudes were evident in the response alternation (12.1 µV/m^2^ ± 1.7) compared to the response repetition condition (10.4 µV/m^2^ ± 1.6). Furthermore, there was a main effect of “finger compatibility” [F(1,27) = 9.75; p = 0.004; *η*_*p*_^2^ = 0.265]. Alternating the stimulated finger resulted in larger amplitudes (12.1 µV/m^2^ ± 1.6) than finger repetition (10.3 µV/m^2^ ± 1.7). The interaction of “response x finger compatibility” was not significant [F(1,27) = 0.42; p = 0.524; *η*_*p*_^2^ = 0.015]. Analysis of the P3 ERP revealed a main effect of “response” [F(1,27) = 35.69; p < 0.001; *η*_*p*_^2^ = 0.569]. Amplitudes were larger in the response repetition (11.5 µV/m^2^ ± 1.5) than in the response alternation condition (8.8 µV/m^2^ ± 1.6). The main effect of “finger compatibility” was not significant [F(1,27) = 1.48; p = 0.235; *η*_*p*_^2^ = 0.052]. The interaction of “response x finger compatibility” was significant [F(1,27) = 7.63; p = 0.010; *η*_*p*_^2^ = 0.220]. Post-hoc paired t-tests demonstrated that repetition and alternation of the stimulated finger resulted in significantly different P3 amplitudes when the response was alternated [t(27) = -2.66; p = 0.013], but not when the response was repeated [t(27) = 0.79; p = 0.436]. In case of response alternation, amplitudes were smaller when the same finger was stimulated (7.7 µV/m^2^ ± 1.5) than when finger stimulation was alternated (10 µV/m^2^ ± 1.8). Post-hoc paired t-tests also showed that response repetition and alternation differed when the same finger was repeatedly stimulated [t(27) = 5.01; p < 0.001] as well as when the stimulated finger alternated [t(27) = 2.26; p = 0.032]. To summarize, no interaction that is consistent with the behavioral interaction effect can be obtained based on standard ERPs.

### Residue iteration decomposition (RIDE)

**Figure 3:**
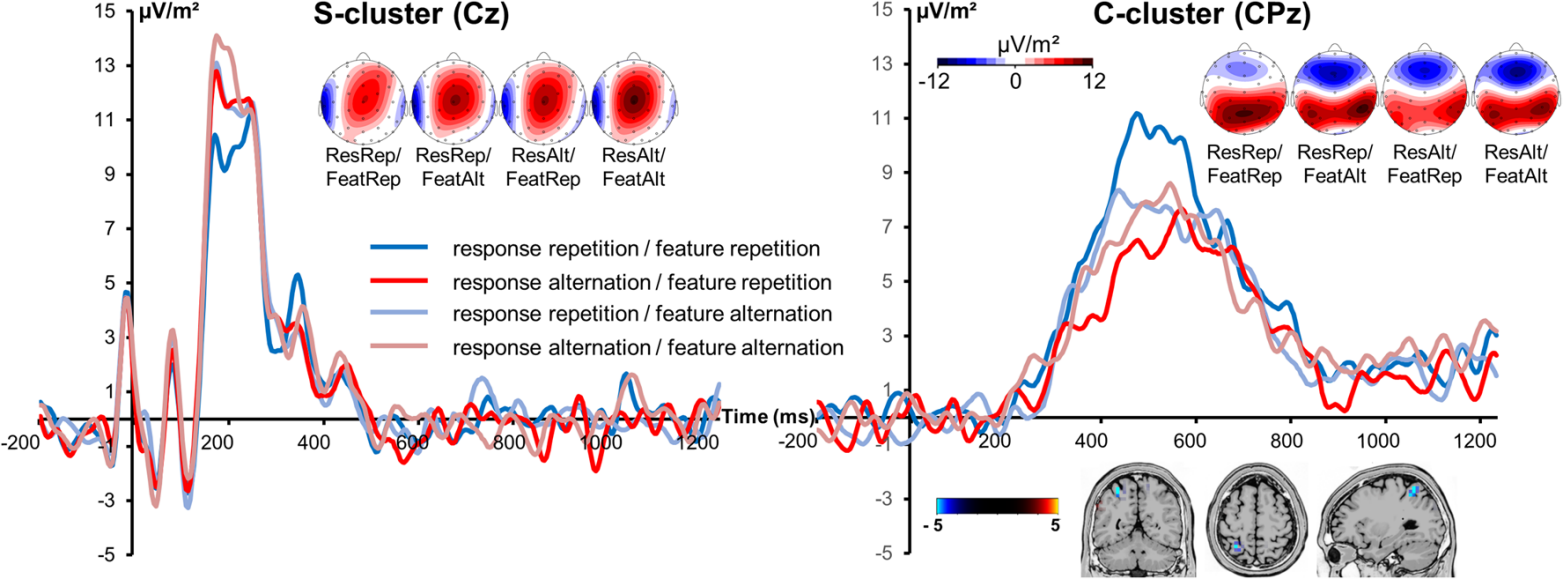
The S-cluster at electrode Cz (left) and the C-cluster at electrode CPz (right). Time point 0 marks S2 stimulus presentation. Different colors of the components represent different conditions (ResRep/FeatRep = response repetition/feature (finger) repetition; ResRep/FeatAlt = response repetition/feature (finger) alternation; ResAlt/FeatRep = response alternation/feature (finger) repetition; ResAlt/FeatAlt = response alternation/feature (finger) alternation) according to the legend. Scalp topography plots show the S- and C-cluster in the different conditions with red illustrating positive and blue negative values. The sLORETA plots illustrate differences in C-cluster modulations of feature (finger) repetition and feature alternation between the response repetition and response alternation condition. The corresponding color scale represents critical t values (corrected for multiple comparisons).

The S-cluster is shown in Fig. 3.

The repeated measures ANOVA for the S-cluster in the P2 time window revealed a main effect of “response” [F(1,27) = 9.02; p = 0.006; *η*_*p*_^2^ = 0.250]. Amplitudes were larger when responses were alternated (12 µV/m^2^ ± 1.7) than when they were repeated (10.2 µV/m^2^ ± 1.6). Moreover, a main effect of “finger compatibility” was found [F(1,27) = 9.35; p = 0.005; *η*_*p*_^2^ = 0.257]. In case the stimulated finger was alternated, larger amplitudes were evident (12 µV/m^2^ ± 1.6) compared to the condition when the same finger was stimulated repeatedly (10.2 µV/m2 ± 1.7). No interaction effect paralleling the relevant behavioral interaction was found.

In the P3 time window the repeated measures ANOVA of the C-cluster showed a main effect of “response” [F(1,27) = 6.08; p = 0.020; *η*_*p*_^2^ = 0.184]. Response repetition was associated with larger C-cluster amplitudes (9.1 µV/m^2^ ± 1.2) than response alternation (7.2 µV/m^2^ ± 1.4). The main effect of “finger compatibility” was not significant [F(1,27) = 0.57; p = 0.459; *η*_*p*_^2^ = 0.020] Furthermore, there was an interaction of “response x finger compatibility” [F(1,27) = 9.91; p = 0.004; *η*_*p*_^2^ = 0.268]. Post-hoc paired t-tests revealed a significant difference between repetition and alternation of the stimulated finger when the response was repeated [t(27) = 2.63; p = 0.014], but not when the response was alternated [t(27) = − 1.16; p = 0.256]. In case of response repetition, larger amplitudes occurred when the same finger was repeatedly stimulated (10.6 µV/m^2^ ± 1.4) than when it was alternated (7.6 µV/m^2^ ± 1.3). This interaction is consistent with the interaction found in the behavioral data and is illustrated in Fig. 3. The sLORETA analysis showed that differences between the conditions were associated with activation differences in the left superior parietal cortex (BA 7).

Moreover, post-hoc paired t-tests showed that response repetition and alternation differed when the same finger was repeatedly stimulated [t(27) = 3.93; p = 0.001] but not when the stimulated finger was alternated [t(27) = − 0.36; p = 0.725]. Under these circumstances, smaller amplitudes were evident when the response was alternated (6.5 µV/m^2^ ± 1.4) than when it was repeated (10.6 µV/m^2^ ± 1.4).

**Figure 4:**
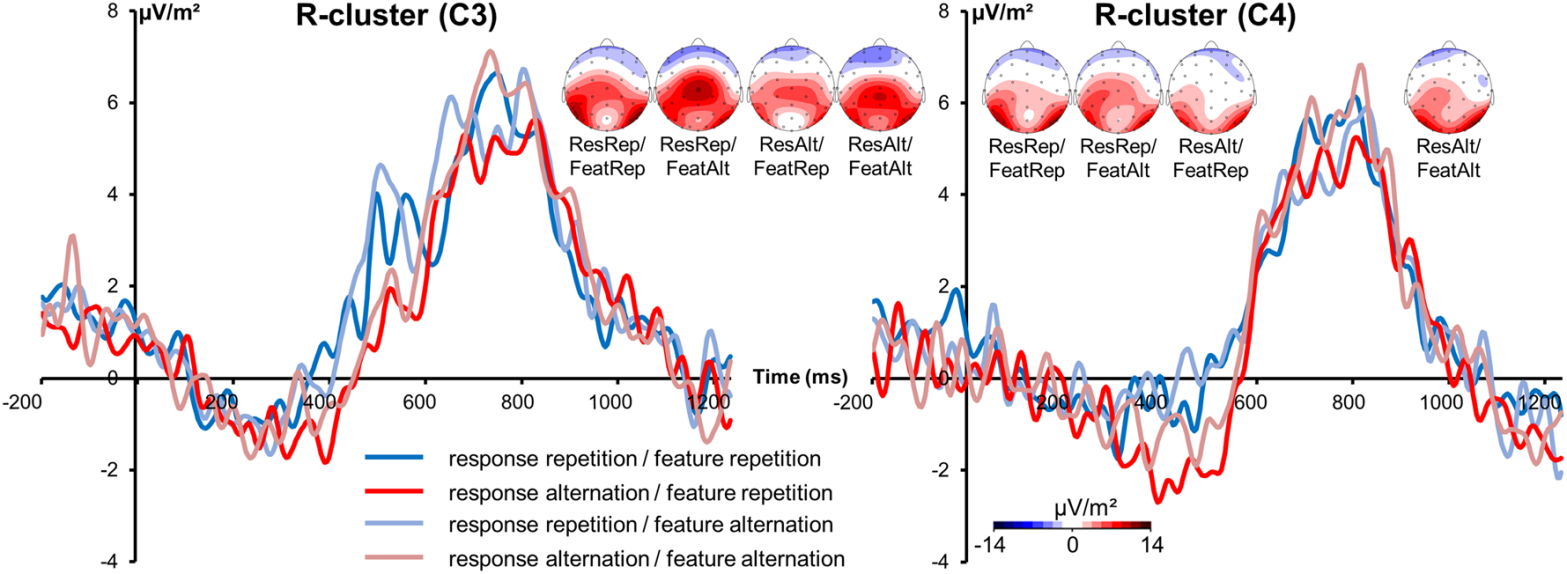
The R-cluster at electrode C3 (left) and electrode C4 (right). Time point 0 marks S2 stimulus presentation. Different colors of the components represent different conditions (ResRep/FeatRep = response repetition/feature (finger) repetition; ResRep/FeatAlt = response repetition/feature (finger) alternation; ResAlt/FeatRep = response alternation/feature (finger) repetition; ResAlt/FeatAlt = response alternation/feature (finger) alternation) according to the legend. Scalp topography plots show the R-cluster in the different conditions with red and blue indicating positive and negative values, respectively.

The R-cluster is shown in Fig. 4.

Repeated measures ANOVA for the R-cluster at electrodes C3 and C4 revealed no significant main or interaction effects (all F ≤ 2.32; p ≥ 0.140). The R-cluster therefore does not reflect the interaction found on the behavioral level.

### Possible effect of perceptual threshold

We used linear regression to test for a potential influence of the individual perception thresholds, thresholds of unpleasant perception and stimulation intensity on stimulus–response binding effects reported above. For this purpose, the difference between the performance in the finger repetition and finger alternation condition was calculated separately for the response repetition and the response alternation condition. Subsequently, these differences were included separately as dependent variables, whereas the different psychophysical measures (perception threshold, threshold of unpleasant perception and stimulation intensity) were used as predictors, respectively. Correspondingly, six models were computed. Linear regression models showed no significant effects (all F ≤ 0.79; p ≥ 0.545).

## Discussion

The present study investigates the neurophysiological basis (as indicated by event-related potentials) of stimulus–response binding in the somatosensory modality. For this purpose, EEG recording was combined with signal decomposition and source localization analysis to identify the neurophysiological underpinnings of somatosensory-motor event file binding in the TEC framework. We expected binding effects akin to binding during visuo-motor processing but also distinctive features.

Behavioral results clearly demonstrated binding between stimulus and response features using somatosensory, i.e., tactile information as perceptual input. When responses had to be repeated, behavioral performance, reflected in reaction time and the inverse efficiency score, was superior when the stimulus feature (finger) was repeated as compared to its alternation, demonstrating repetition benefits. These results can be interpreted along the lines of a pattern-completion logic^3–5^: In conditions of response repetition, re-encountering a specific stimulus feature automatically activates previously established links, improving task performance. Encountering an alternated stimulus feature in turn automatically triggers activation of another response, not corresponding to the required one. In this case, the event file has to be reconfigured, impairing task performance. Thus, in keeping with previous data, a feature repetition benefit was present in response repetition conditions^8,12,17^. In contrast, when the response was alternated, there was no performance difference between feature repetition or alternation, indicating a lack of repetition costs. This finding differs from studies in the visual domain typically showing both repetition benefits in case of feature overlap when the response was repeated and repetition costs when the response was alternated^2,8,12,39^.

Given the premise that repetition benefits and costs are based on the same mechanisms, the question arises as to why the former but not the latter was present during somatosensory-motor event file binding. One possibility is that binding, as tested with the current experimental setup, is weaker compared to visuo-motor binding, perhaps because the (electrical) stimuli used are biologically less meaningful or salient than typically used visual stimuli. If so, re-activation of the previously established event file is less likely to interfere with current task demands, neither in a disadvantageous nor in a beneficial way under the condition of response alternation. This assumption can be examined in more detail taking neurophysiological results into account.

Behavioral results were paralleled by neurophysiological (ERP) findings, but only after applying EEG signal decomposition confirming the benefit of RIDE application. This was expected since neurophysiological effects have already been found to be more pronounced based on a decomposed EEG signal in the visual domain^12^. In addition, distractor-response binding effects were evident at a neurophysiological (i.e., event-related potential) level using temporally decomposed ERP data^11^. The reason is that the traditional approach to average ERP components results in a composite of information originating from different sources. Particularly in the P3 time window, there are different components reflecting stimulus- and response-related processes^26^, which is likely due to the high latency variability of the P3 component^29–31^. Moreover, RIDE minimizes intra-individual variability by the application of L1-norm estimation for ERP decomposition, whereas traditional ERP averaging methods rely on reducing the L2-norm. This type of data processing is more prone to intra-individual variability^29,31,40^.

After RIDE application, it was revealed that not stimulus-related but rather stimulus–response transition processes, as indicated by C-cluster modulations, accounted for behavioral findings during event file processing. In contrast, S- and R-clusters were not affected by modulations of stimulus–response binding, suggesting that object and action files remain unchanged when event files are modified^3^, which is also in line with neurophysiological results found for the visual domain^11,12^. As expected, also in the present study, stimulus-related processing reflected by the S-cluster did not parallel the interaction found at the behavioral level. The P2 component associated with the allocation of attentional resources during the processing of sensory stimuli^35,36^ did not reveal any interaction for the factors “response x feature (finger) compatibility”. This shows that modulations of behavioral performance cannot be attributed to differences in attentional processing. Differences between conditions manifest beyond the stimulus processing level as has been shown before^20,22,23,25^. Effects seem to be protracted to the stimulus–response transition level as reflected by C-cluster modulations in the P3 time window.

The study conducted in the visual domain^12^ associated smaller C-cluster amplitudes in the P3 time window with better behavioral performance under the condition of joint feature and response repetition. This means that superior behavioral performance was paralleled by a smaller C-cluster amplitude. The present study revealed a larger amplitude in case of feature overlap compared to no feature overlap in the response repetition condition indicating that superior behavioral performance was accompanied by larger C-cluster amplitudes. It can be summarized that repetition benefits are evident in the visual as well as the somatosensory domain, yet underlying neurophysiological processes are modulated in opposite ways. These discrepancies point to differences between the visual and somatosensory domain with respect to perception–action integration processes. Based on findings of smaller P3 amplitudes indicating more difficult response selection processes^41,42^, Takacs and colleagues interpreted smaller C-cluster amplitudes in case of feature overlap and response repetition as successful intensification of response selection processes. Following this logic for the somatosensory modality, larger amplitudes suggest an attenuation of response selection processes. This is well in line with the above-mentioned assumption of weaker stimulus–response binding in the somatosensory domain, since response selection processes are likely to be attenuated when the link between stimulus and associated response is weakened. This notwithstanding, neurophysiological processes seem to be sufficiently modulated to result in superior performance under conditions of feature and response repetition as compared to feature alternation and response repetition. Takacs and colleagues also found that no amplitude modulations occurred in the response alternation condition. Based on the above-mentioned line of argumentation, they interpreted that response selection processes are not sufficiently modulated in case of response alternation. This neurophysiological result is also confirmed by the present experiment since no C-cluster amplitude modulation was evident. It can be interpreted that in case of response alternation, underlying neurophysiological processes reflecting response selection processes are not sufficiently modulated to result in superior performance during feature alternation as it was demonstrated for feature and response repetition. However, it can be interpreted that response selection processes are intensified under conditions of feature repetition and response alternation because there is no drop in performance (i.e., repetition costs) as it is the case in the visual domain. A possible interpretation is that the assumed weakened binding in the somatosensory modality and the associated attenuated response selection process as demonstrated for repetition benefits has a protective function with respect to repetition costs. The weakened binding probably requires reduced event file reconfiguration processes under feature repetition and response alternation conditions so that no repetition costs arise. Taken together, similar to the visual modality, it is presumably more likely that amplitude modulations are associated with the success of response selection than indicating the amount of event file re-activation^12^. It can be concluded that different neurophysiological processes inherent to the different modalities can lead to comparable behavioral outcomes (i.e., repetition benefits). It has to be noted that the paradigms used in the visual and somatosensory modalities differ significantly, so that a direct transfer of underlying neural mechanisms is not possible.

Source localization analyses (sLORETA) revealed that differences between the binding effects in the response repetition and response alternation conditions were associated with activation differences in the left superior parietal cortex (BA 7). The smaller C-cluster amplitude difference between the finger repetition and finger alternation condition in case of response alternation was accompanied by less activation in the left superior parietal cortex. Conversely, the larger difference between the finger repetition and finger alternation condition in case of response repetition was correlated with stronger activation in this area. BA 7 has already been linked to C-cluster modulations in the context of action control using vibro-tactile stimuli^22,23^. Activation of this area is plausible given that the superior parietal cortex as part of the somatosensory association cortex located in the posterior parietal cortex (PPC) receives direct input from primary somatosensory areas^43^. Furthermore, it is convincing to associate this area with stimulus–response binding since it has been shown that the PPC is a processing structure not exclusively for purely sensory or motor information^44,45^ but rather the integration of a large variety of information (e.g., visual, somatosensory and motor)^46,47^. Importantly, the superior parietal cortex (BA 7) has been shown to be engaged in the integration of somatosensory with action-related information^46,48^, which is crucial during event file formation. Therefore, it is unlikely that PPC activation is merely a consequence of right hand stimulation. Nevertheless, future studies should investigate whether bilateral stimulation results in deviating activation patterns. However, it should be noted that switching the stimulation site is a potential confound, as it might be included as a feature in the event file. Stronger activation of BA 7 associated with larger amplitudes and superior behavioral performance likely reflects sufficient modulation of response selection processes based on efficient sensory and motor information integration. Less activation in this area, however, is rather accompanied by insufficient recruitment of response selection capacities likely due to impaired stimulus–response integration.

Pulse features of the stimuli did not modify stimulus–response binding. It has already been demonstrated that learning determines whether a specific stimulus feature is bound into an event file^49^. It has been shown that with increasing task practice, the binding of stimulus features that are not task-relevant decreases. Since only the finger dimension was relevant to complete the task successfully, this is presumably the reason that pulse features did not affect event file binding.

Linear regression models showed that different perceptual thresholds as well as differences in stimulation intensity have no predictive value for stimulus–response binding effects. Obviously, “lower-level” perceptual processes do not affect event file binding processes. Studies in the somatosensory domain using vibro-tactile stimuli have already found that, although basic perceptual processes were manipulated, effects manifest at the response selection level in the context of action control^20,22,23,25^. The fact that the variation of sensory processes affects event file binding by modulating response selection processes can be explained considering the TEC. Since stimulus- and response-related information as well as links established between them are stored in an event file^2,3^, the effects of the variation of sensory processes can protract to the response selection level. However, the event file is not supposed to reflect basic properties of lower-level perception per se but rather the link or associations that are formed between perceptual and action-related features. Stimulus-related information, like stimulus modality, is stored in the object file^1^.

To summarize, the present study examined the neurophysiological underpinnings of event file binding in the somatosensory modality using EEG signal decomposition and source localization. Behavioral repetition benefits were evident when responses were repeated. However, in contrast to findings in the visual domain, no repetition costs emerged when responses were alternated indicating differences in event file binding mechanisms between different modalities. EEG signal decomposition showed C-cluster modulations reflecting repetition benefits evident at the behavioral level. This corroborates the theory-derived hypothesis of event files reflecting stimulus–response linkage. Larger C-cluster amplitudes associated with superior behavioral performance likely reflect efficient recruitment of response selection resources required for presumably weakened stimulus–response linkage. This is associated with stronger integration of sensory and motor information as reflected by superior left parietal cortex activation likely facilitating response selection processes. Due to modality differences, results found in the visual domain cannot be directly transferred to the somatosensory domain. Nevertheless, TEC still provides a useful framework to explain behavioral and neurophysiological findings in the context of somatosensory-motor event file binding.

## Materials and methods

### Participants

N = 28 healthy subjects (16 females) between the age of 19 to 30 years (mean age = 24; SEM = 0.61) were examined in this experiment. The participants reported no psychiatric or neurological disorders and confirmed right-handedness. The subjects signed a written informed consent prior to the experiment and all performed methods were in line with relevant guidelines and regulations. The local ethics committee of the Medical Faculty of the TU Dresden and University of Lübeck authorized this study.

### Procedure and task

**Figure 5:**
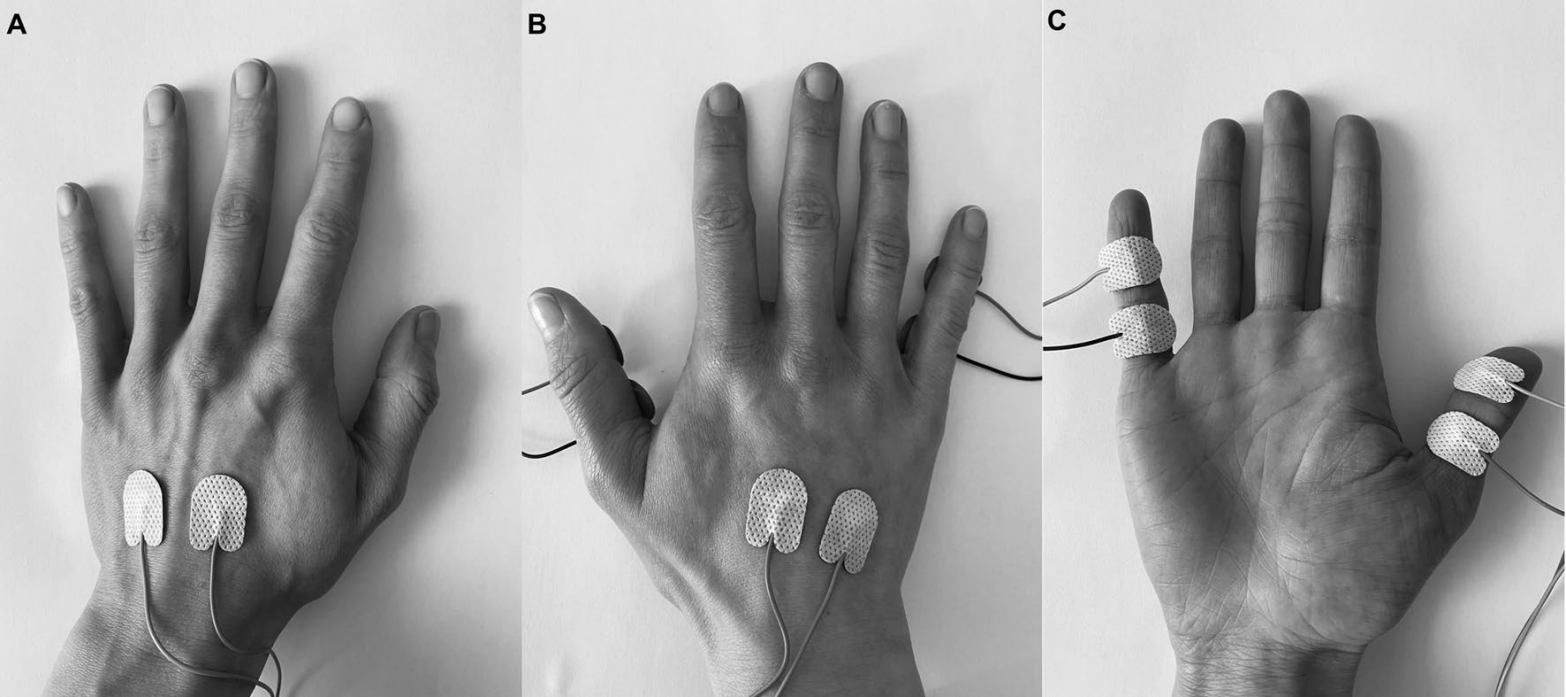
Illustration of the experimental setup. Surface adhesive electrodes were attached at a distance of approximately 1 cm to the back of the left hand (**A**), the back of the right hand (**B**), and the thumb and little finger of the right hand (**C**). Participants were required to respond to electrotactile stimuli by pressing the left or right control key with their left or right index finger, respectively.

To investigate somatosensory-motor event file binding, a tactile version of the stimulus–response (S-R) task developed by Colzato et al.^8^ was conducted. Electro-tactile stimuli (unipolar positive electrical pulses, duration 0.2 ms, 300 V) were applied using eight disposable surface adhesive electrodes (two at each site fixated at a distance of approximately 1 cm from each other) attached to the back of both hands and the palmar side of the thumb and little finger of the right hand. Stimulation was generated by an ISIS Neurostimulator (Inomed, Emmendingen, Germany; https://www.inomed.de/), controlled by experimental software developed in Python 2.7 using the expyriment toolbox^50^. The experimental setup is shown in Fig. 5.

The design of the (electro-tactile) somatosensory-motor S-R task we used was adapted from the original S-R task in the visual modality^8^.

In the tactile version used in the current study, a cue (a single electrical pulse) was administered to the back of the left or right hand. After an interval of 2,500 ms, the first stimulus (S1) was given either to the thumb or to the little finger of the right hand. S1 consisted of either two or four electrical pulses (for details please see below). As soon as the S1 was given, participants were asked to indicate whether the cue had been given to the back of their left or right hand by pressing the left or right control key with their left or right index finger regardless of the stimulus features of S1. In case the response was incorrect or too slow (i.e., not within a time range of 500 ms after S1 presentation ended), the word ’Wiederholung’ (German word for ’repetition’) was displayed for 500 ms and the trial was repeated up to three times. 2,500 ms after offset of S1, the second stimulus (S2), consisting of two or four pulses, was applied to the thumb or little finger of the right hand. In response to S2, participants were asked to press the left control key with their left index finger when stimulation was administered to the thumb or the right control key with their right index finger when stimulation was delivered to the little finger. Reactions occurring within a time window of 2000 ms after S2 offset were registered. The inter-trial interval was jittered between 1,500 to 2000 ms.

Consequently, there were different compatibility conditions. Either all stimulus features were compatible (i.e., application of the same pulse sequence to the same finger as S1 and S2), only one feature was compatible (i.e., either the same pulse sequence or stimulation at the same finger) or no feature was compatible (i.e., different pulse sequence and finger). In addition, the two reactions, i.e., the reaction to the cue during S1 and the reaction to S2, were either identical or differed. This setting could lead to repetition benefits or repetition costs. Each condition occurred equally across the experiment. In case responses are repeated, task performance usually improves with the number of compatible stimulus features (repetition benefits), whereas response repetition deteriorates performance the more features are incompatible (repetition costs)^8,10^. Repetition or alternation of all features (stimulus and response) has a positive effect on performance compared to the partial repetition of features/response. The latter is commonly referred to as ‘partial-repetition cost’^1,3,5^.

**Figure 6:**
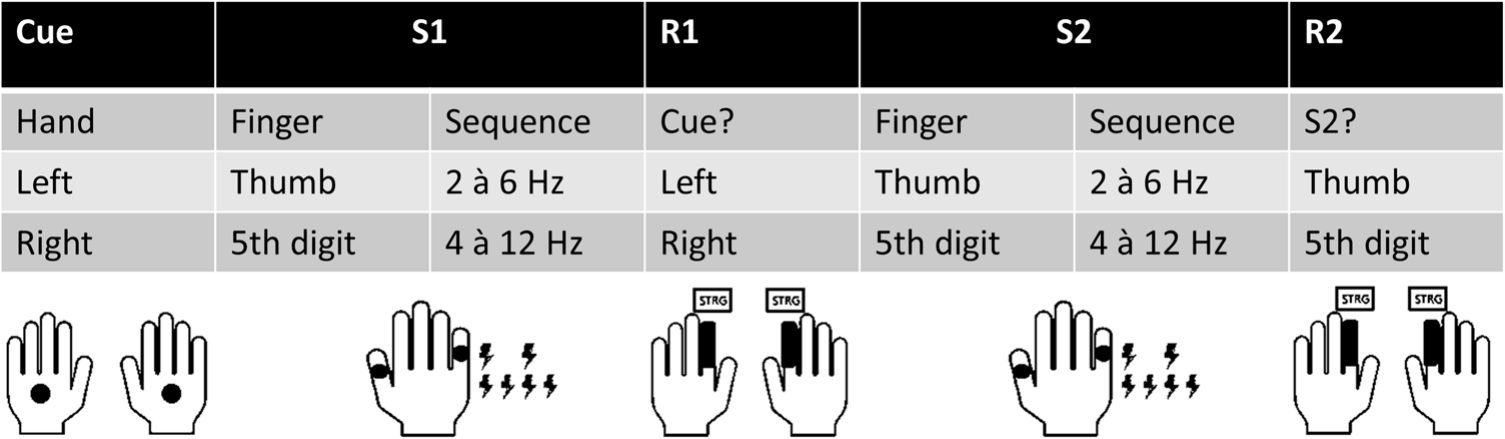
Schematic illustration of the experiment. Each black dot represents a pair of electrodes. Stimuli (S1, S2) and responses (R1, R2) are shown in chronological order. The exact temporal course is described in the text.

Individual electrical pulses all had a duration of 0.2 ms. The stimulus dimensions finger (thumb vs. little finger) and pulse number (double or four pulses) were chosen because they allowed a clear differentiation of stimulus features. This is also the reason why no adjacent fingers were used. S1 and S2 electrical pulse series were applied such that the total duration of the stimulation period in the different conditions was comparable. In the double pulse condition, two pulses at a frequency of 6 Hz were given, i.e., these pulses were given within a period of 167 ms. In the quadruple pulse condition, four pulses at frequency of 12 Hz were applied, i.e. the stimulation period amounted to 250 ms. The schematic illustration of the experimental setup can be found in Fig. 6. A total of 384 trials was tested, divided into six blocks. Conditions occurred equally within the blocks. The sequence of conditions was randomized so participants were unable to predict stimulus type or stimulation site. Prior to the experiment, a practice block was run comprising 16 trials. Trial onset was always indicated on the screen as ’Next trial’ (’Nächster Durchgang’ in German) and displayed for 500 ms.

### Measurement of the perception threshold

Individual perception thresholds were determined for each stimulation site separately (back of the left and right hand, thumb and little finger) prior to experimental testing using single pulses (unipolar positive 300 V pulses delivered for 0.2 ms). Starting at an intensity of 1 mA, the participants were asked to indicate whether or not an electrical pulse was perceived. If a given pulse was not perceived, stimulus intensity was gradually increased by 0.5 mA until the pulse was clearly perceived. Next, intensity was increased by 2 mA so that it was clearly supra-threshold. Starting from this intensity, stimulus intensity was decreased stepwise using the same step size as in the ascending series. Participants were again required to indicate if the administered pulse was perceived. This was continued until the stimulation was no longer felt. Subsequently, this procedure, i.e., an ascending followed by a descending series was repeated. After perception thresholds were determined, we then defined thresholds where stimulation was considered discomforting as follows. Starting at the mean perception threshold, stimulation intensity was raised in steps of 1 mA until the participant labelled the administered pulse as clearly discomforting. The mean of the perception threshold and the threshold for unpleasant perception was used as the stimulation intensity in the experiments. Participants were asked if pulses given at these intensities were clearly perceptible and not unpleasant. In a final step, stimulation intensities were compared between both backs of the hands and the fingers and intensity was adapted to the stronger stimulus until stimulation at both sites felt equally intense. To investigate whether tactile sensitivity changes during the course of the experiment, preliminary studies were conducted with additional threshold measurements between the experimental blocks. No changes in perception threshold could be detected in this respect.

### EEG recording and analysis

60 passive Ag/AgCl ring electrodes, arranged at equidistant positions and connected to a QuickAmp amplifier (Brain Products Inc.) were used to record the EEG. Pre-processing of recorded data was accomplished with BrainVision Analyzer (Brain Products Inc.). Ground and reference electrodes were positioned at coordinates theta = 58, phi = 78 and theta = 90, phi = 90, respectively. A sampling rate of 500 Hz was used to record EEG data while electrode impedances remained below 5 kΩ and the recoding bandwidth was set to 0.5–80 Hz. A down-sampling to 256 Hz was then carried out after recording as well as the application of an IR band-pass filter set to 0.5–20 Hz with a slope of 48 dB/oct. To account for irregular technical or muscular artefacts a manual raw data inspection was performed. Frequently recurring artefacts caused by blinking or moving the eyes laterally were addressed by an independent component analysis (ICA; infomax algorithm) carried out for all blocks. Identified independent components clearly representing artefacts were then discarded prior to the segmentation of data performed for each experimental condition separately. A segment started 2000 ms before S2 stimulus presentation and ended 2000 ms after it. Segments were locked to stimulus onset. Only trials in which all responses were correct were included in the analysis. This means that the correct response to the cue had to occur within 500 ms after the end of the presentation of S1 and the correct response to S2 had to occur within 2000 ms after the end of S2 presentation. Next, an automated artefact rejection was run to eliminate trials with a maximal value difference of 200 μV in a 200 ms period. Amplitudes below − 200 μV and above 200 μV and below 0.5 μV in a 100 ms time window were also defined as rejection criteria. This step was followed by current source density (CSD) transformation using 4 splines and 10 polynomials. Applying a CSD transformation allows for reference-free assessment of the data by removing the reference potential. This procedure results in amplitude values in μV/m^2^. The benefit of this transformation is that it facilitates the identification of relevant electrodes for subsequent data quantification since it operates as a spatial filter (Nunez & Pilgreen, 1991; Tenke & Kayser, 2012). Subsequently, a baseline correction from -200 to 0 was performed with time point 0 reflecting S2 stimulus presentation. Then the averages of the different conditions (no compatibility/compatibility of stimulus features between S1 and S2 and response repetition/alternation) were calculated and quantification of ERP amplitudes was conducted on a single-subject level. On this basis, grand averages were computed separately for the different conditions. The P2 component was clearly evident at electrode Cz and was quantified as the mean amplitude in the time period between 140 and 190 ms for all conditions. The P3 component was evident at electrode CPz and quantified in the time window between 450 to 550 ms for all conditions. The decision about relevant time windows and electrode sites was made after the visual inspection of related scalp topography maps.

### Residue iteration decomposition

The RIDE algorithm was run with MATLAB (MATLAB 12.0; Mathworks Inc.) using EEG data on the single-trial level. For this purpose, the RIDE toolbox was applied (available on https://cns.hkbu.edu.hk/RIDE.htm) in line with previous work^26,29,51^. The mathematical details of the applied algorithm can be found in previous literature on this method^52^.

The principle idea is that the residual error caused by latency variability of ERPs in single trials is minimized^52^. To this end, the RIDE procedure decomposes ERP components into different clusters^30^. This is accomplished for each electrode separately without considering scalp distributions or waveforms so that performing the CSD transformation does not distort results. The benefit of applying this algorithm is the decomposition of ERPs into clusters that are associated with stimulus onset (S-cluster), response times (R-cluster) and an intermediate cluster occurring between stimulus and response with variable latency (C-cluster). In an initial step, the C-cluster waveform is estimated which subsequently undergoes iterative improvements. For C-cluster latency estimation, a time window function is applied which is then subjected to a self-optimizing iteration scheme used to enhance estimated C-cluster latency. For this purpose, the S-cluster is removed and the C-cluster re-estimated using a template matching approach. An important step refers to the predefinition of the time intervals, in which clusters are assumed to occur. These time periods need to be adapted to the data of the respective study^52^. For more details, please refer to Ouyang et al.^29,31,52^. The S-cluster was expected to occur in the time range between − 200 to 500 ms around stimulus onset and the C-cluster in a time window between 250 and 900 ms in approximate accordance with previous studies^20,23^. The time interval between − 300 to 300 ms was assumed to cover the R-cluster linked to the response time. The S-cluster was quantified at the same electrode (i.e., Cz) and in the same time interval (i.e., 140 to 190 ms) as the standard P2 ERP component for all conditions. This also applies to the C-cluster that was quantified in accordance with the P3 component at electrode CPz in the time window between 450 and 550 ms. The C-cluster has already been associated with processes linked to the P3 component like stimulus–response transition^26,32,53^. The R-cluster was quantified at electrodes C3 and C4 depending on the mean reaction times in the different conditions (± 50 ms). Accordingly, the time window between 505 to 605 ms was quantified in the response repetition/feature (finger) repetition condition. In the response repetition/feature (finger) alternation condition the time period between 523 to 623 ms was chosen and for the response alternation/feature (finger) repetition condition the time window was set at 514 to 614 ms. In case of response alternation/feature (finger) alternation the time interval between 508 and 608 ms was quantified.

### Source localization analysis

Source localization analysis was implemented by means of sLORETA (standardized low resolution brain electromagnetic tomography)^54^. sLORETA provides the benefit that a single solution to the inverse problem is supplied^54–56^. Initially, the intra-cerebral volume is partitioned into 6,239 voxels with a respective spatial resolution of 5 mm. Subsequently, the standardized current density is quantified for each voxel based on an MNI152 template^57^ using a realistic three-shell spherical head model^58^. It was shown mathematically that sLORETA provides reliable results without localization bias^56^. Moreover, EEG/fMRI and neuronavigated EEG/TMS studies confirmed the sources identified by sLORETA^56,59^. The sLORETA-built-in voxel-wise randomization tests with 2000 permutations working on the basis of statistical nonparametric mapping (SnPM) were used to compare voxel-based sLORETA images across conditions (i.e., to compare binding effects in the response repetition and response alternation condition). Voxels expressing significant differences (p < 0.01, corrected for multiple comparisons) between the computed contrasts of interest were presented in the MNI brain.

### Statistical analysis

Behavioral results (i.e., accuracy rates, reaction times in correct trials and inverse efficiency index) were evaluated using repeated measures analyses of variance (ANOVAs). Three within-subject factors were defined: “feature (finger) compatibility” (stimulation applied twice to the same (feature repetition) or to alternating fingers (feature alternation)), “pulse compatibility” (stimulation with the same/alternating pulse sequence) and “response” (response repetition/alternation). Since the factor “pulse compatibility” showed no significant interaction with the ”response” factor, which is crucial to demonstrate stimulus–response binding effects, it was not included in the analyses of neurophysiological data. Possible reasons for the lack of “pulse compatibility” effects are discussed above. Neurophysiological data was analyzed using repeated measures ANOVAs with the two within-subject factors “finger compatibility” and “response”. For R-cluster analysis, the factor “electrode” (C3/C4) was added as a within-subject factor. Greenhouse–Geisser correction was applied to all tests and Bonferroni correction was used for post-hoc tests. Mean values and standard errors of the mean (SEM) are given in brackets in the following “Results” section.

## Data Availability

The datasets generated during and/or analyzed during the current study are available from the corresponding author on reasonable request.
